# Supplementary material for: Osmolar Modulation Drives Reversible Cell Cycle Exit and Human Pluripotent Cell Differentiation via NF‐κВ and WNT Signaling
Source: Adv Sci (Weinh). 2023 Dec 1;11(7):2307554. doi: 10.1002/advs.202307554 (PMC10870039; doi:10.1002/advs.202307554)
Supplement: Supplementary file 1 — Supporting Information [file ADVS-11-2307554-s002.pdf]

ADVANCED SCIENCE Open Access

Supporting Information

for *Adv. Sci.*, DOI 10.1002/advs.202307554

Osmolar Modulation Drives Reversible Cell Cycle Exit and Human Pluripotent Cell Differentiation via NF-κB and WNT Signaling

*Jonathan Sai-Hong Chui, Teresa Izuel-Idoype, Alessandra Qualizza, Rita Pires de Almeida, Lindsey Piessens, Bernard K. van der Veer, Gert Vanmarcke, Aneta Malesa, Paraskevi Athanasouli, Ruben Boon, Joris Vriens, Leo van Grunsven, Kian Peng Koh, Catherine M. Verfaillie and Frederic Lluis**

Supporting Information

Osmolar modulation drives reversible cell cycle exit and human pluripotent cell differentiation via NF-κB and WNT signaling

Jonathan Sai-Hong Chui[1], Teresa Izuel-Idoype[1]*, Alessandra Qualizza[1], Rita Pires de Almeida[1], Lindsey Piessens[1], Bernard K. van der Veer[1], Gert Vanmarcke[1], Aneta Malesa[1], Paraskevi Athanasouli[1], Ruben Boon[1], Joris Vriens[2], Leo van Grunsven[3], Kian Peng Koh[1], Catherine M. Verfaillie[1#], Frederic Lluis[1#&]*

[1]KU Leuven, Department of Development and Regeneration, Stem Cell Institute, B-3000, Leuven, Belgium

[2]Laboratory of Endometrium, Endometriosis and Reproductive Medicine, Department of Development and Regeneration, KU Leuven, Leuven, Belgium

[3]Liver Cell Biology Research Group, Vrije Universiteit Brussel, Laarbeeklaan 103, 1090, Brussels, Belgium.

* These authors contributed equally

#Co-last authors.

[&]Email: Frederic.lluisvinas@kuleuven.be

<u>**Supplementary Figures**</u>

Supplementary Figure 1

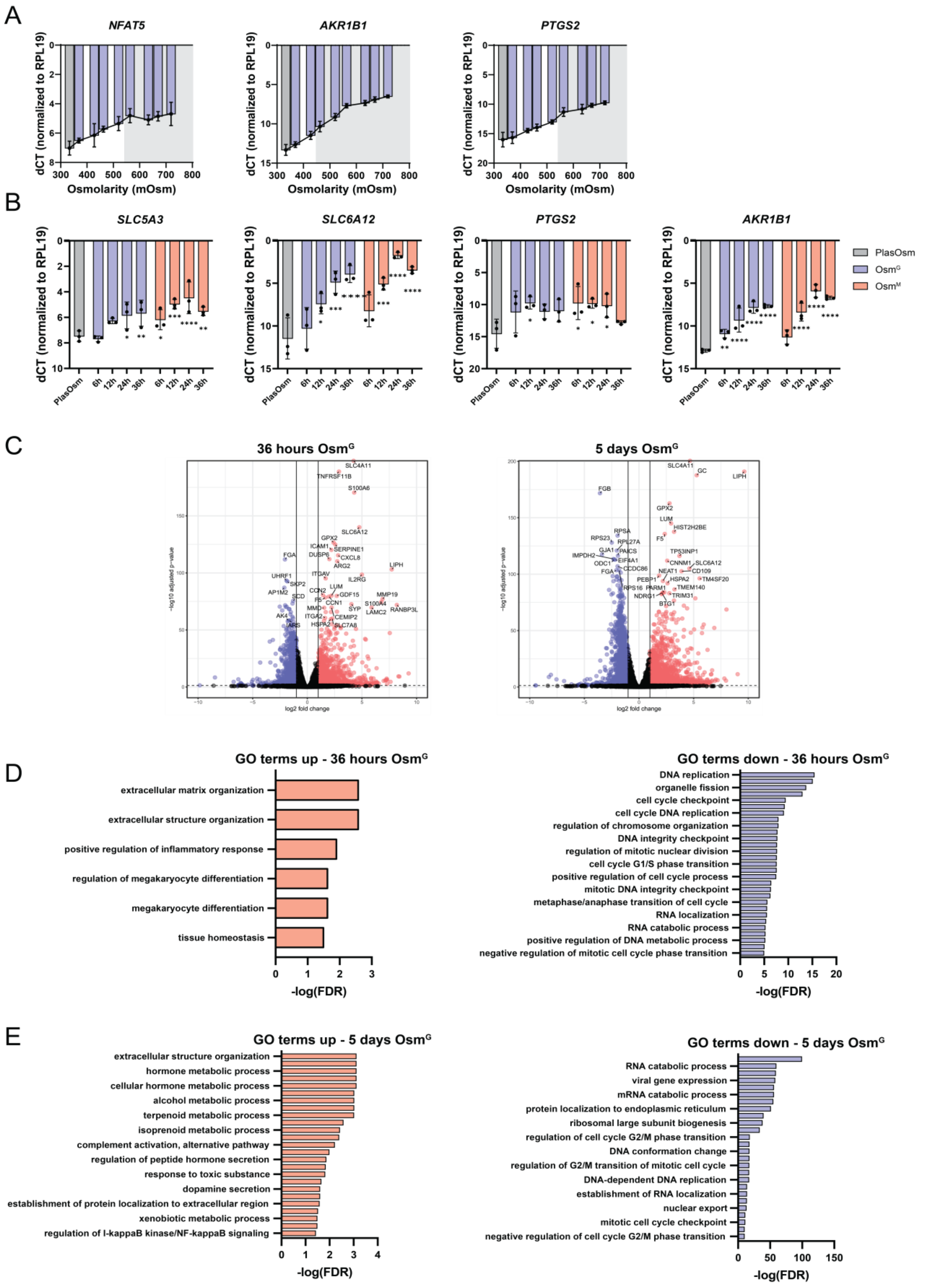

Supplementary Figure 1:

- **(A)** Gene expression of NFAT5 target genes detected by RT-PCR normalized to housekeeping gene *RPL19* across different levels of hyperosmolarity under Osm^G. Statistical significance was determined compared to the control condition. N=3, statistics by Two-way ANOVA.
- **(B)** Gene expression of osmo-adaptive genes detected by RT-PCR normalized to housekeeping gene RPL19 measured across different time points of hyperosmolarity (550 mOsm) under Osm^G and Osm^M treatment. Statistical significance was determined compared to the control condition. N=3, statistics by Brown-Forsythe and Welch ANOVA.
- **(C)** Volcano plots representing up- and downregulated DEGs upon 36 hours and 5 days of Osm^G treatment compared to PlasOsm conditions. Full list of DEGs shown in Supplementary Table 4.
- **(D)** Following cut-off values of FDR 0.05 and a fold change of 2, Differentially Expressed Genes were enriched for Gene Ontology terms after 36 hours of treatment. Full list of GO terms shown in Supplementary Table 5.
- **(E)** Following cut-off values of FDR 0.05 and a fold change of 2, Differentially Expressed Genes were enriched for Gene Ontology terms after 5 days of treatment. Full list of GO terms shown in Supplementary Table 5.

Supplementary Figure 2

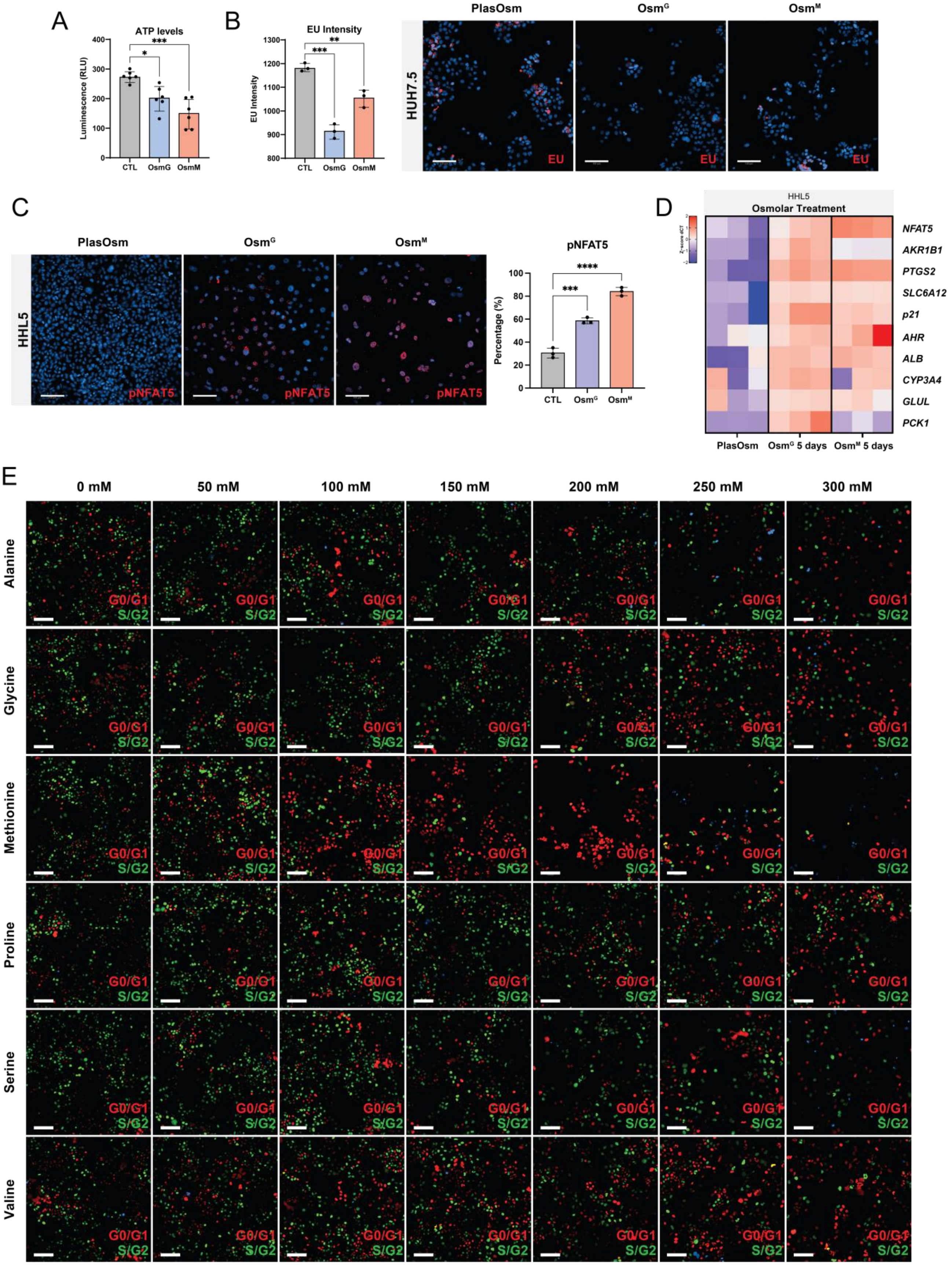

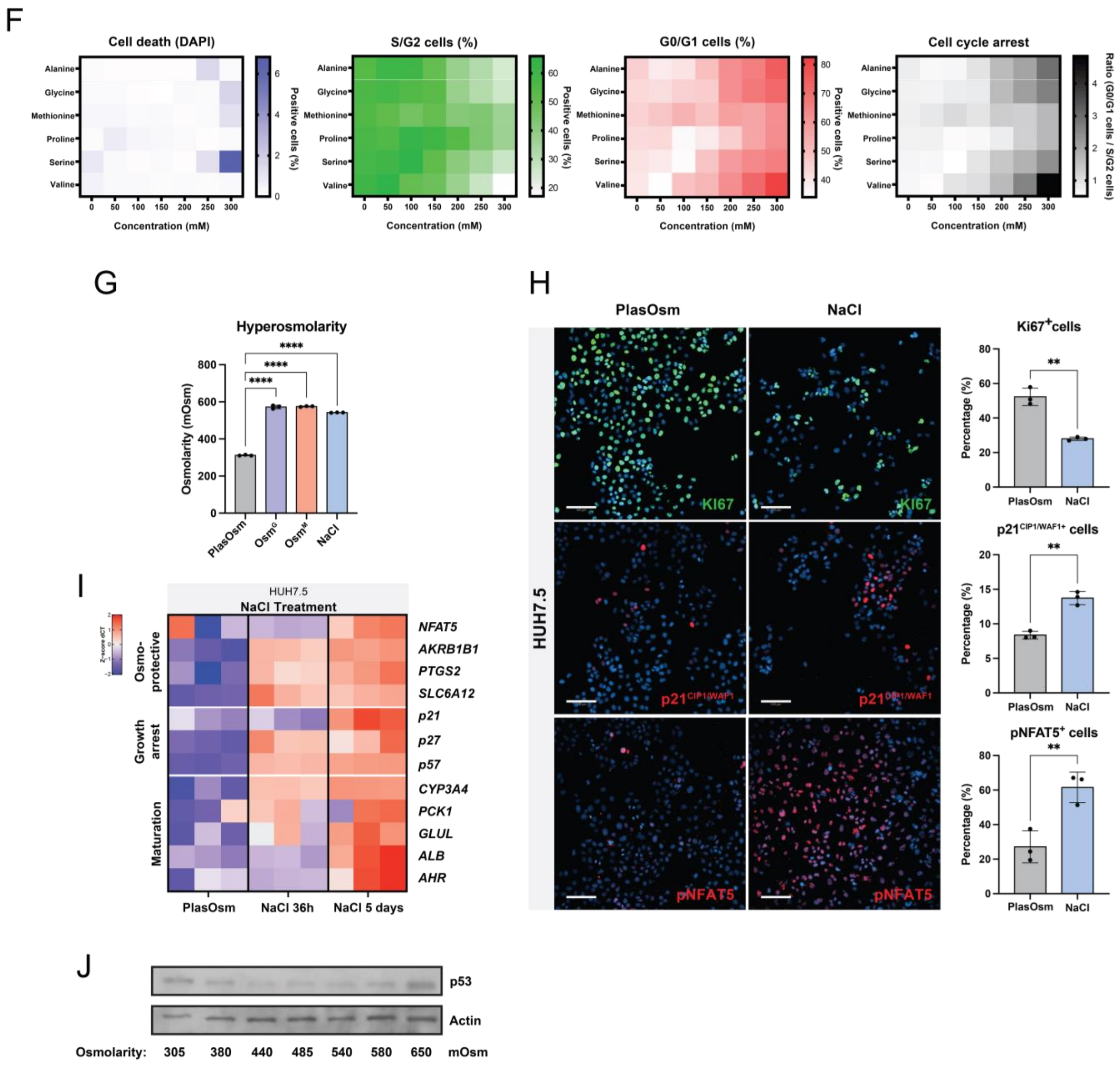

Supplementary Figure 2:

- **(A)** Luminescent detection of ATP levels after 1 hour of Osm^{G} and Osm^{M} treatment. N=3, statistics by Brown-Forsythe and Welch ANOVA.
- **(B)** Immunofluorescence (right) and quantification (left) of EU incorportation in HUH7.5 cells treated with Osm^{G}and Osm^{M} for 24 hours. Scale bar = 100 µm. N=3, statistics by Brown-Forsythe and Welch ANOVA.
- **(C)** Immunofluorescence staining (left) of NFAT5 upon Osm^{G} and Osm^{M} treatment in HHL5 cells with quantification of nuclear detection (right). N=3, statistics performed by Brown-Forsythe and Welch ANOVA tests. Scale Bar = 100µm.
- **(D)** Gene expression analysis of osmo-protective (*NFAT5, AKR1B1, PTGS2, SLC6A12*), growth arrest (p21$^{CIP1/WAF1}$) and hepatic maturation (*AHR, ALB, CYP3A4, GLUL, PCK1*) genes after 5 days of treatment with Osm^{G} and Osm^{M} in HHL5 cells normalized to *RPL19* expression and visualized in z-score of the dCT values. N=3.
- **(E)** Confocal images of FUCCI-HUH7.5 cells treated with increasing concentrations of indicated amino acids (up). Scale bar = 100 µm.
- **(F)** Quantification of cell death (DAPI), cells in S/G$_2$ phase, cells in G$_0$/G$_1$ phase and cells in cell cycle arrest represented in heatmaps (down).
- **(G)** Osmolarity levels of culture medium (PlasOsm) supplemented with NaCl (125 mM) shown in comparison with Osm^{G} and Osm^{M} culture conditions represented in mOsm. N=3, statistics by Ordinary one-way ANOVA

(H) Immunoflurescent detection and quantification of KI67, $p21^{CIP1/WAF1}$ and NFAT5 in HUH7.5 cells after 24 hours in medium supplemented with 125 mM NaCl. Scale bar = 100 µm. N=3, statistics by Brown-Forsythe and Welch ANOVA.

(I) Gene expression analysis of osmo-protective, growth arrest and hepatic maturation genes after 36h and 5 days of treatment with 125 mM NaCl normalized to *RPL19* expression and visualized in z-score of the dCT values. N=3.

(J) Western blot showing the protein levels of p53 and Actin following Osm^M treatment for 36 hours with increasing osmolarity levels.

Supplementary Figure 3

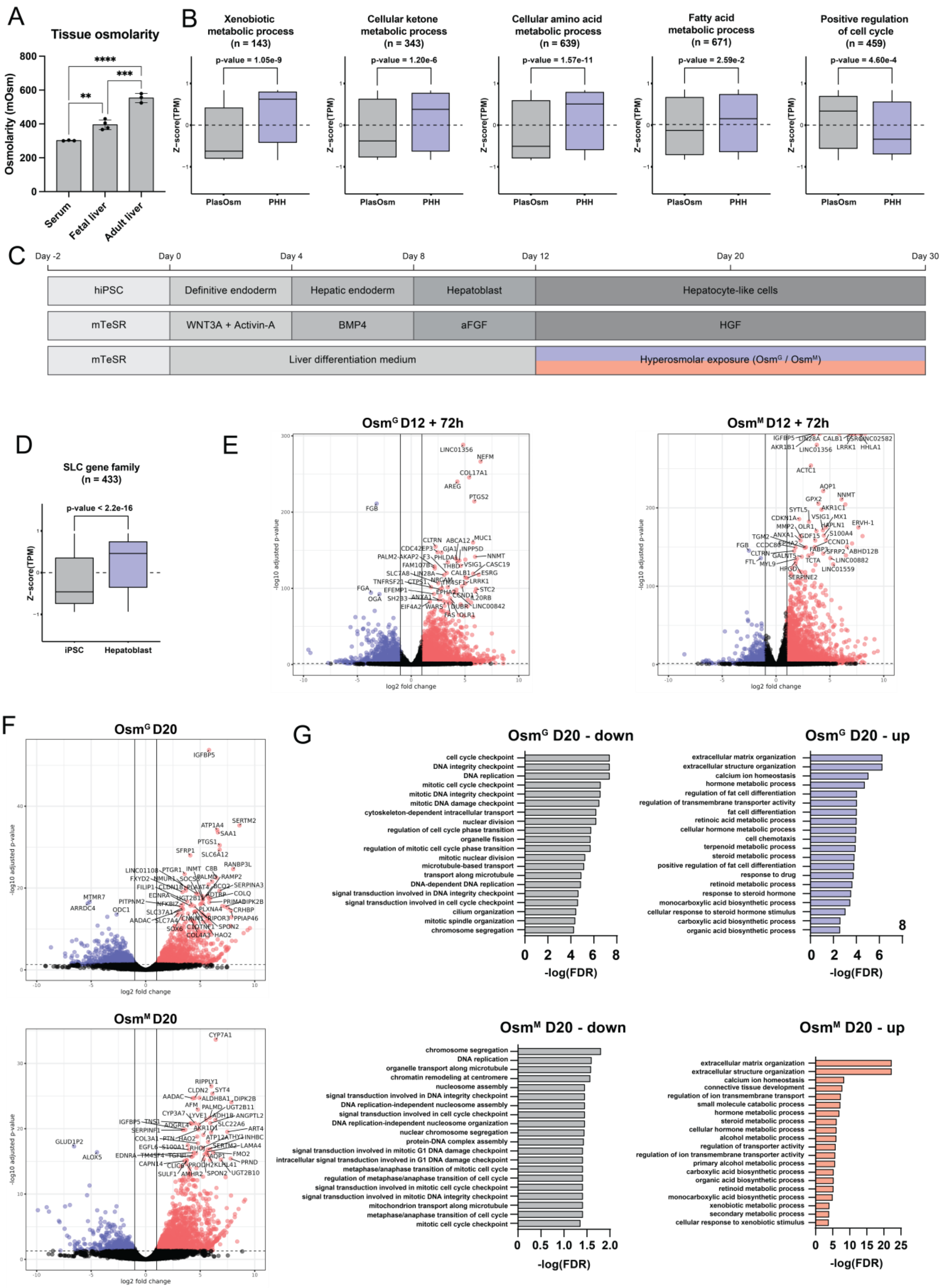

H

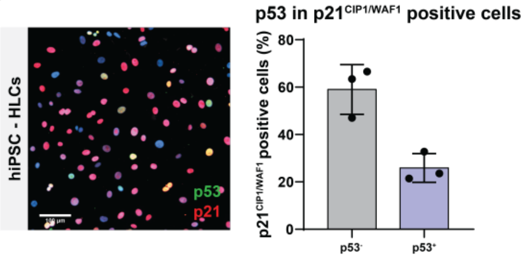

I

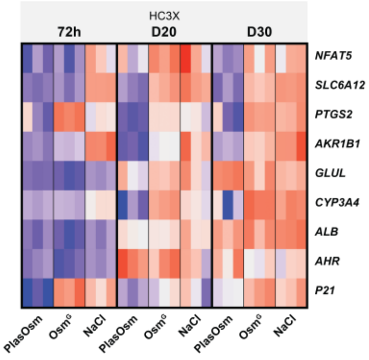

J

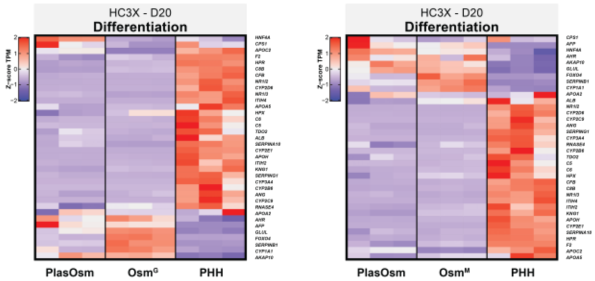

Supplementary Figure 3:

- **(A)** Tissue osmolarity levels measured in liver tissues collected from fetal mice (E13.5) and adult mice (10 weeks) represented in mOsm and compared to serum levels shown in Fig. 1B). N=3 for serum and adult liver, N=4 for fetal liver, statistics by Brown-Forsythe and Welch ANOVA.
- **(B)** Boxplots representing the expression of the gene sets of specific gene ontology (GO) terms represented in the z-score of TPM values. Statistics by Mann-Whitney test. N = number of genes represented within each GO term and is indicated on represented boxplots. Full list of genes shown in Supplementary Table 6.
- **(C)** Visual representation of the growth factor-based hepatic differentiation from induced PSCs[34].
- **(D)** Boxplots representing the expression of the gene sets of SCL gene ontology (GO) family represented in the z-score of TPM values. Statistics by Mann-Whitney test. N = 433 genes in the GO term. Full list of genes shown in (Supplementary Table 6).
- **(E)** Volcano plots showing up (left) and downregulated (right) DEGs upon Osm^{G} and Osm^{M} treatment of D12 iPSC-HLCs after 72 hours.
- **(F)** Volcano plots showing up (top) and downregulated (bottom) DEGs upon Osm^{G} and Osm^{M} treatment of D20 iPSC-HLCs.
- **(G)** Gene Ontology (GO) enrichment of differentially expressed genes (DEGs) comparing PlasOsm vs HypOsm (Osm^{G}) at D20 of differentiation. Full list of GO terms shown in Supplementary Table 8.
- **(H)** Representative image (left) and quantification (right) of fluorescent detection of p53 and p21$^{CIP1/WAF1}$ after 72h of Osm^{G} treatment. Scale bar = 100 µm. N=3, statistics by Two-Way ANOVA.
- **(I)** Gene expression analysis of osmo-protective (NFAT5, AKR1B1, PTGS2, SLC6A12), growth arrest (p21$^{CIP1/WAF1}$) and hepatic maturation (AHR, ALB, CYP3A4, GLUL) genes after 72 hours of Osm^{G} and NaCl treatment and at day 20 and day 30 of the Osm^{G} and NaCl treated differentiation protocol in iPSC-HLCs cells normalized to *RPL19* expression and visualized in z-score of the dCT values. N=3.
- **(J)** Heatmap of hepatic maturation markers upon Osm^{G} and Osm^{M} treatment in hiPSC-derived HLCs at D20 of differentiation compared to fresh PHHs.

All data represents mean ± SEM; *p < 0.05, **p < 0.01, ***p< 0.001, ****p<0.0001.

Supplementary Figure 4

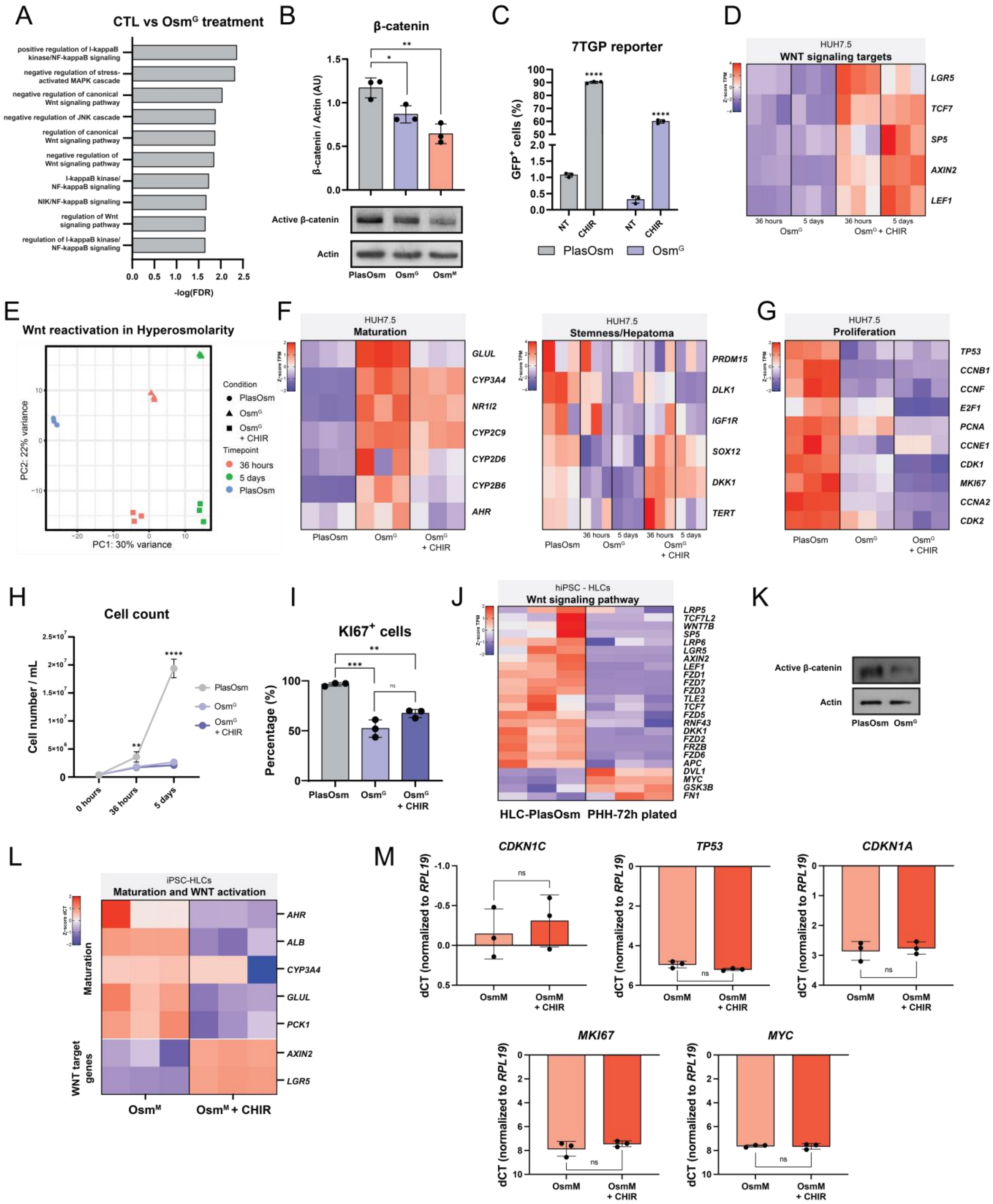

Supplementary Figure 4: Wnt signaling regulates lineage specification during hyperosmolarity guided differentiation.

- **(A)** Gene ontology (GO) enrichment reveals differentially expressed signaling pathways between control and Osm^G treated cells at 36h after treatment. Full list of GO terms shown in Supplementary Table 9.
- **(B)** Western blot of active β-catenin levels (upper panel) visualized and quantified to Actin levels (lower panel) after Osm^G or Osm^M treatment. N=3. Statistics by One-way ANOVA.
- **(C)** Percentage of GFP^+ HUH7.5 cells containing the WNT transcriptional reporter 7TGP treated with CHIR99021 in CTL and Osm^G conditions. N=3. Statistics by One-way ANOVA.
- **(D)** Transcriptional levels of Wnt target genes across Osm^G and Osm^G + CHIR99021. Data is represented in z-score of TPM values.
- **(E)** PCA plot of RNA-seq samples under control, Osm^G and Osm^G + CHIR99021 conditions.
- **(F)** Heatmap of genes involved in maturation (left) and stemness/hepatocarcinoma (right) represented in z-score of TPM values across samples.
- **(G)** Heatmap of genes involved in proliferation represented in z-score of TPM values across samples.
- **(H)** Cell count of hepatoma HUH7.5 cell line at 0 and 36 hours and 5 days after control, Osm^G and Osm^G + CHIR99021 treatment. N=3. Statistics by Two-way ANOVA.
- **(I)** KI67 detection by flow cytometry under Osm^G and Osm^G + CHIR99021 conditions after 36 hours. N=3, statistics by One-way ANOVA.
- **(J)** Heatmap of genes involved in WNT signaling represented in z-score of TPM values across HLCs at D30 in PlasOsm conditions compared to PHHs plated.
- **(K)** Western blot of non-phosphorylated (active) form of b-catenin at D30 of iPSC-HLCs under PlasOsm and Osm^G conditions. Actin was used as an internal loading control across samples.
- **(L)** Gene expression analysis of hepatic maturation markers and WNT target genes after treatment with Osm^M and Osm^M + CHIR99021 at D30 of the differentiation protocol. Data is represented in z-score of the dCT levels. N=3.
- **(M)** Gene expression analysisof genes related to proliferation after treatment with Osm^M and Osm^M + CHIR99021 at D30 of the differentiation protocol. N=3. Statistics by Welch's t-test.

All data represents mean ± SEM; *p < 0.05, **p < 0.01, ***p< 0.001, ****p<0.0001.

Supplementary Figure 5

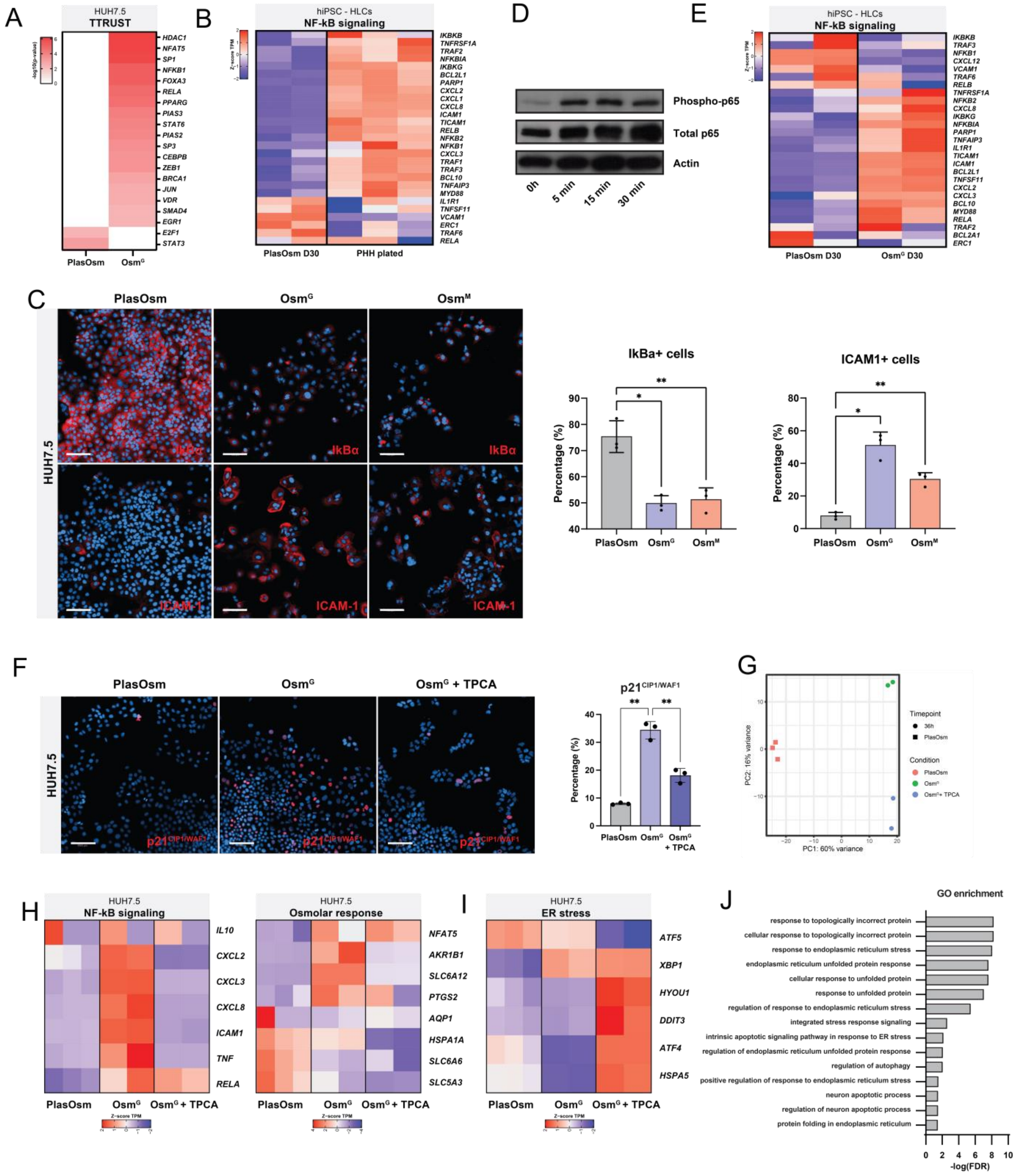

Supplementary Figure 5:

- **(A)** Transcription factor prediction using the TTRUST tool on differentially expressed genes at 36 hours of Osm^G treatment.
- **(B)** Heatmaps of bulk RNA-seq data representing the z-score of TPM values across presented sample conditions showing genes associated with NF-κB signaling comparing hiPSC-HLCs under PlasOsm at day 30 to PHHs.
- **(C)** Representative images (left) and quantification (right) of immunofluorescent detection of IκBα and ICAM-1 under PlasOsm, Osm^G and Osm^M conditions in HUH7.5 cells. Scale bar = 100 µm. N=3, statistics by Brown-Forsythe and Welch ANOVA tests.
- **(D)** Western blot of phosphorilated-p65 and total p65 upon 5 min, 15 min and 30 min of HypOsm (Osm^M) treatment in HUH7.5 cells. Actin was used as a loading control across samples.
- **(E)** Heatmaps of bulk RNA-seq data representing the z-score of TPM values across presented sample conditions showing genes associated with NF-κB signaling comparing hiPSC-HLCs under PlasOsm and Osm^G treatment.
- **(F)** Representative images (left) and quantification (right) of immunofluorescent staining of $p21^{CIP1/WAF1}$ under PlasOsm, Osm^G and Osm^G + TPCA conditions in HUH7.5 cells. Scale bar = 100 µm. N=3, statistics by Brown-Forsythe and Welch ANOVA tests.
- **(G)** Principal component analysis of bulk RNA-seq data of PlasOsm, Osm^G and Osm^G + TPCA treated HUH7.5 cells after 36 hours of treatment.
- **(H)** Heatmaps of bulk RNA-seq data representing the z-score of TPM values across presented sample conditions of HUH7.5 cells showing genes associated with NF-κB signaling and osmolar response.
- **(I)** Heatmaps of bulk RNA-seq data representing the z-score of TPM values across presented sample conditions of HUH7.5 cells showing genes associated with ER stress.
- **(J)** Gene ontology (GO) enrichment of upregulated DEGs in TPCA-1 treated HUH7.5 cells under Osm^G conditions. Full list of GO terms shown in (Supplementary Table 14).

All data represents mean ± SEM; *$p < 0.05$, **$p < 0.01$, ***$p < 0.001$, ****$p < 0.0001$.

Supplementary Figure 6

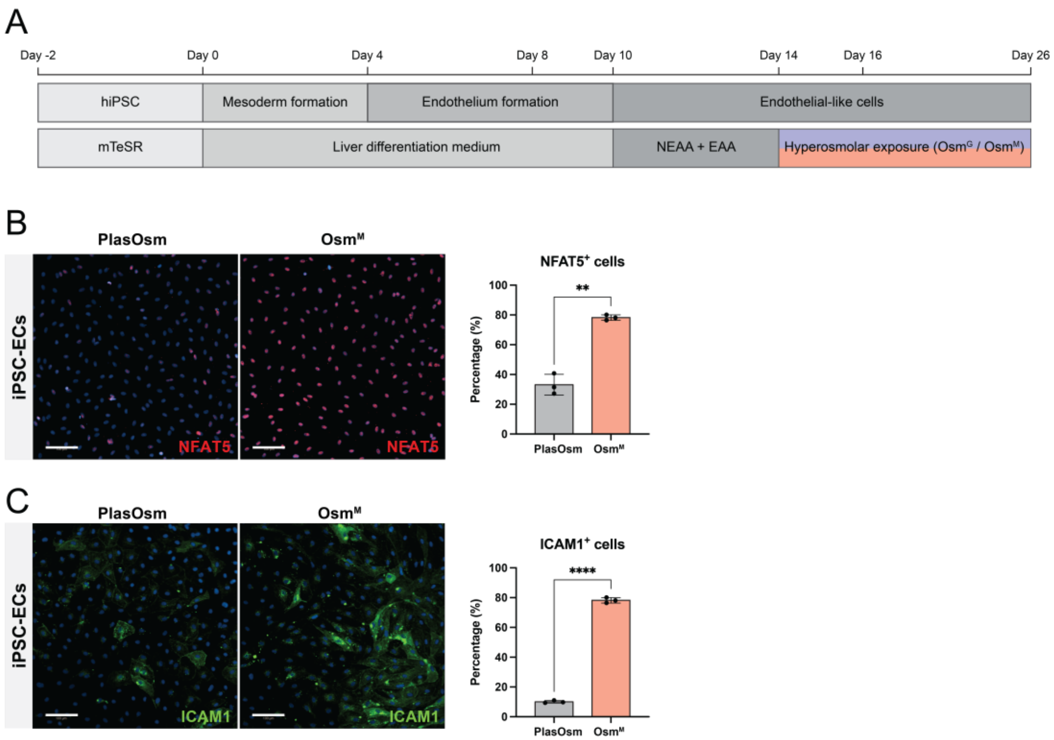

Supplementary Figure 6: The adaptive osmo-protective response is a common mechanism shared by the mesodermal lineage.

- **(A)** Visual representation of the growth factor-based endothelial cell differentiation protocol.
- **(B)** NFAT5 detection by immunofluorescence treated with Osm^M after 48 hours. Statistics performed by t-test with Welch's correction. N=3.
- **(C)** ICAM1 detection by immunofluorescence treated with Osm^M after 48 hours. Statistics performed by t-test with Welch's correction. N=3.

All genes were normalized to housekeeping gene *RPL19*. All scale bars = 100 µm. All data represents mean ± SEM; *p < 0.05, **p < 0.01, ***p< 0.001, ****p<0.0001. Controls depicted in this supplementary figure are the same as in Figure 7.

Supplementary Information – Western Blot scans

Figure 2J

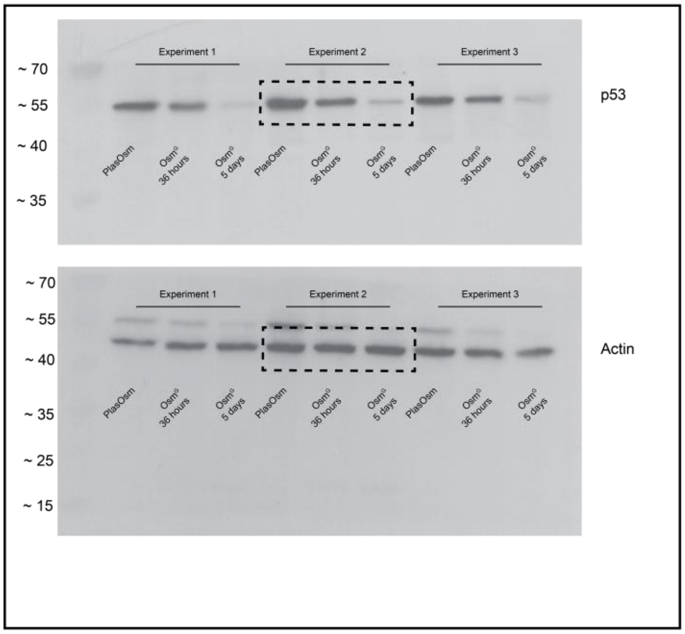

Figure S2J

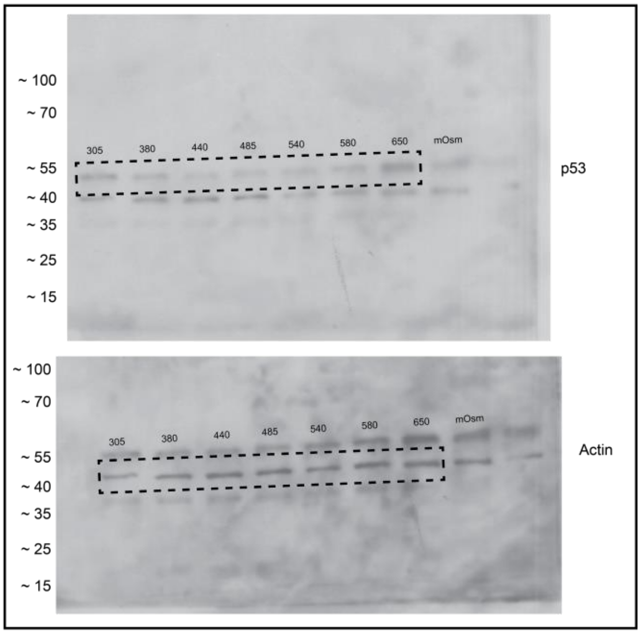

Figure S4B

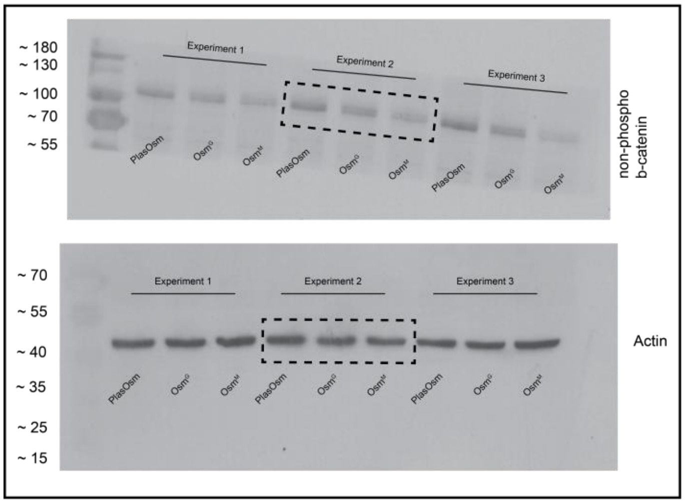

Figure S5D

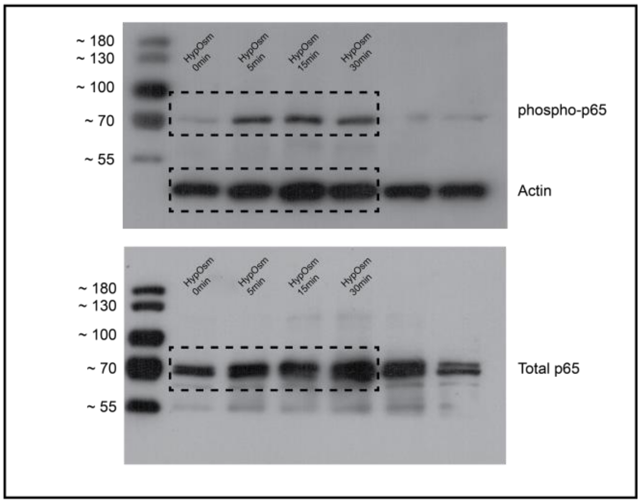

Figure S4K

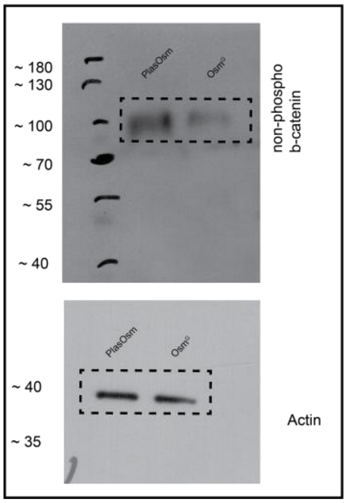

Supplementary Tables

Supplementary methods – Reagents table

Product	Company	Catalog number
DMEM low glucose	Gibco	31885023
Fetal Bovine Serum (FBS)	Thermo (Life tech)	10270-106
Pen-strep	Gibco	15070063
Trypsin-EDTA 0.25%	Gibco	15050065
Sigma 0028	Sigma-Aldrich	IPSC0028
Corning™ Matrigel™ hESC-Qualified Matrix	Corning	734-1440
Matrigel Based Membrane Growth Factor Reduced	Corning	734-0270
Essential 8™ Flex Medium Kit	Gibco	A2858501
Ethylenediaminetetraacetic acid (EDTA)	Thermo (Life Technologies)	15575020
PBS, pH 7.4	Gibco	10010023
StemPro Accutase	Sigma-Aldrich	A6964
mTeSR™1 Complete Kit	Stem Cell Technologies	85850
RevitaCell™ Supplement (100X)	Gibco	A2644501
WNT-3A	Peprotech	315-20
Activin A	Peprotech	120-14E
BMP4	Peprotech	120-05ET
acidic FGF	Peprotech	120-05ET
HGF	Peprotech	100-39
DMSO	Merck	D2650
bFGF	Peprotech	100-18C
Doxycycline	Sigma-Aldrich	D9891
Endothelial Cell Growth Supplement (ECGS) (50X)	BioTechne - R&D systems	390599
Glycine	Sigma-Aldrich	1041691000
Mannitol	Sigma-Aldrich	M4125
MEM Amino Acids Solution (50X)	Gibco	11130051
MEM Non-Essential Amino Acids Solution (100X)	Gibco	11140050
PFA	Sigma-Aldrich	158127
Triton-X	Sigma-Aldrich	T8787
Donkey serum	Jackson Immunoresearch	017-000-121
DAPI	Sigma-Aldrich	D9542
annexin V binding buffer	BD Pharmigen	51-66121E
APC-conjugated annexin	Thermo-eBioscience	BMS306APC-100
Click-iT™ EdU Alexa Fluor™ 647 Flow Cytometry Assay Kit	ThermoFisher	C10418
7-Benzyloxy-4-trifluoromethylcoumarin (BFC)	Merck	B5057-5MG
AFP Human ELISA Kit	Invitrogen	EHAFP
Albumin ELISA Kit	Bethyl Laboratories, Inc.	BET E80-129
QuantiChrom Urea Assay Kit	BioAssay Systems	DIUR-100
GenElute Mammalian Total RNA Miniprep Kit	Merck (Sigma)	RTN350-1KT
iScript cDNA synthesis kit	Bio-Rad	1708891
KAPA stranded mRNA HyperPrep kit (96rxn)	Roche	KK8581
KAPA-single index adapters	Roche	KK8702
Agentcourt AMPure XP beads	Beckman Coulter	A63880
High Sensitivity QuBit kit	Invitrogen	Q33230

CellTiter-Glo 3D Cell Viability Assay	Promega	G9681
GloMax Discover	Promega	SA4000
5-Ethynyl-uridine	Jena Bioscience	CLK-N002-10

Table 1 - Antibody list.

**Antibody**	**Catalog number**	**Company**	**Dilution**
Phospho-NFAT5-Ser155	STJ90634	St John's Laboratory	1:200
Anti-phospho-Histone H2A.X (Ser139)	05-636	Merck	1:200
Cleaved Caspase-3 (Asp175) (5A1E)	9664	Cell signaling Technologies	1:200
Ki-67 recombinant mAb (SP6)	MA5-14520	Invitrogen	1:50
p21 Waf1/Cip1 (12D1)	2947S	Cell signaling Technologies	1:200
p53 (DO-1)	sc-126	Santa Cruz	1:200
Non-phospho (Active) β-Catenin (Ser45) (D2U8Y)	19807S	Cell signaling Technologies	1:200
Cyp3a4	BS90368	bioworld	1:200
Total B-catenin	610154	BD Biosciences	1:200
IκBα (L35A5) (Amino-terminal Antigen)	4814S	Cell signaling Technologies	1:200
ICAM 1 (15.2)	sc-107	Santa Cruz	1:200
LYVE-1	HPA042953	Sigma	1:200
Ki-67	556003	BD Biosciences	1:200
Phospho-NF-κB p65 (Ser536) (93H1) Rabbit mAb	3033T	Cell signaling Technologies	1:1000
NF-κB p65 (D14E12) XP® Rabbit mAb	8242S	Cell signaling Technologies	1:1000

Table 2 - Primer list.

**Gene**	**5'-3' sequence**	**3'-5' sequence**
RPL19	AGTATGCTCAGGCTTCAGAAGA	ATTGGTCTCATTGGGGTCTAAC
CYP3A4	TTCCTCCCTGAAAGATTCAGC	GTTGAAGAAGTCCTCCTAAGCT
ALB	TGGCACAATGAAGTGGGTAA	CTGAGCAAAGGCAATCAACA
AHR	GTCGTCTAAGGTGTCTGCTGGA	CGCAAACAAAGCCAACTGAGGTG
PCK1	CATTGCCTGGATGAAGTTTGACG	GGGTTGGTCTTCACTGAAGTCC
SLC5A3	GCCAGTACCATATTCACCCTCG	CATCTCCACGATGATTGGCACC
SLC6A12	CATCTCCACGATGATTGGCACC	GCTGAACAGGTAGAAGAGAGCC
PTGS2	CGGTGAAACTCTGGCTAGACAG	GCAAACCGTAGATGCTCAGGGA
AKR1B1	CCAACTTCAACCATCTCCAGGTG	GTCACCACGATGCCTTTGGACT
GLUL	CTGCCATACCAACTTCAGCACC	ATAGGCACGGATGTGGTACTGG
CDKN1A	AGGTGGACCTGGAGACTCTCAG	TCCTCTTGGAGAAGATCAGCCG
CDKN1B	ATAAGGAAGCGACCTGCAACCG	TTCTTGGGCGTCTGCTCCACAG
CDKN1C	AGATCAGCGCCTGAGAAGTCGT	TCGGGGCTCTTTGGGCTCTAAA
CDKN2A	CTCGTGCTGATGCTACTGAGGA	GGTCGGCGCAGTTGGGCTCC
CDKN2B	ACGGAGTCAACCGTTTCGGGAG	GGTCGGGTGAGAGTGGCAGG
CDKN2C	CGTCAATGCACAAAATGGATTTGG	GAATGACAGCGAAACCAGTTCGG
CDKN2D	GTGCATCCCGACGCCCTCAAC	TGGCACCTTGCTTCAGCAGCTC
MKI67	AAGCCCTCCAGCTCCTAGTC	TCCGAAGCACCACTTCTTCT

CCNE1	TGTGTCCTGGATGTTGACTGCC	CTCTATGTCGCACCACTGATACC
TP53	ACTTCATCTCTCACATCTTAGCCT	AAACAGCGAGCCTCTGGAACCT
MYC	TGAGGAGACACCGCCCAC	CAACATCGATTTCTTCCTCATCTTC
LYVE-1	TTTGCAGCCTATTGTTACAACTCAT	GGGATGCCACCCAGTAGGTA
KDR	ACAACCAGACGGACAGTGGT	AGCCTTCAGATGCCACAGAC
LGR5	CTTACGTCACTGATGGTGCTTGC	CTTGGAGAAAGAGATTTAGCCAGG
AXIN2	AGTGCAAACTTTCGCCAACC	TGAAGGACCTGTATCCACTGTC
CXCL8	GAGAGTGATTGAGAGTGGACCAC	CACAACCCTCTGCACCCAGTTT

Table 3 - Defined gene sets used in heatmaps.

Table 4 - DEGs HUH7.5 PlasOsm vs OsmG.

Table 5 - GO terms HUH7.5 PlasOsm vs OsmG.

Table 6 - Gene lists defined by GO terms used in heatmaps.

Table 7 - DEGs HLC PlasOsm vs OsmG/OsmM.

Table 8 - GO terms HLC PlasOsm vs OsmG/OsmM.

Table 9 - Identified signaling pathways based on DEGs HUH7.5 36h.

Table 10 - PC axes HUH7.5 OsmG vs OsmG + CHIR.

Table 11 - TTRUST enrichment HUH7.5 OsmG 36h.

Table 12 - DEGs HUH7.5 OsmG vs OsmG + TPCA36h.

Table 13 - DEGs HLC OsmG vs OsmG + TPCA 72h.

Table 14 - GO terms HUH7.5 OsmG vs OsmG + TPCA 36h.
